# Antimicrobial resistance patterns of *Salmonella* spp. identified from retail pork and raw side salads from Busia County, Kenya

**DOI:** 10.1093/trstmh/trag019

**Published:** 2026-03-06

**Authors:** Christine Makena Mbabu, Cianjo M Gichuyia, Peter Gathura, James Mbaria, Gitahi Nduhiu, Eric M Fèvre, Lian F Thomas

**Affiliations:** Department of Public Health, Pharmacology and Toxicology; Faculty of Veterinary Medicine, University of Nairobi, PO Box 29053-00625, Nairobi, Kenya; International Livestock Research Institute, PO Box 30709, Nairobi 00100, Kenya; Department of Public Health, Pharmacology and Toxicology; Faculty of Veterinary Medicine, University of Nairobi, PO Box 29053-00625, Nairobi, Kenya; International Livestock Research Institute, PO Box 30709, Nairobi 00100, Kenya; Department of Public Health, Pharmacology and Toxicology; Faculty of Veterinary Medicine, University of Nairobi, PO Box 29053-00625, Nairobi, Kenya; Department of Public Health, Pharmacology and Toxicology; Faculty of Veterinary Medicine, University of Nairobi, PO Box 29053-00625, Nairobi, Kenya; Department of Public Health, Pharmacology and Toxicology; Faculty of Veterinary Medicine, University of Nairobi, PO Box 29053-00625, Nairobi, Kenya; International Livestock Research Institute, PO Box 30709, Nairobi 00100, Kenya; Department of Livestock and One Health, Institute of Infection, Veterinary and Ecological Sciences, University of Liverpool, Liverpool L69 3BX, UK; International Livestock Research Institute, PO Box 30709, Nairobi 00100, Kenya; Royal (Dick) School of Veterinary Studies, Easter Bush Campus, Edinburgh, Midlothian EH259RG, UK

**Keywords:** antimicrobial resistance, Kenya, multidrug resistance, pork, *Salmonella* spp, side salads

## Abstract

**Background:**

Consumption of food contaminated with antimicrobial-resistant (AMR) *Salmonella* spp. poses significant public health risks.

**Methods:**

We investigated AMR patterns in non-typhoidal *Salmonella* (NTS) isolated from raw pork, ready-to-eat pork and side salads from butcheries in Busia County, Kenya (April–October 2019). A total of 81 isolates underwent antimicrobial susceptibility testing using disc diffusion according to Clinical and Laboratory Standards Institute 2018 guidelines.

**Results:**

Of the isolates, 90% (73/81) were resistant to at least one antibiotic, 22.2% were multidrug resistant and 9.8% exhibited extended drug resistance. High resistance was observed to gentamicin (70.4%), ampicillin (38.3%) and cefuroxime (37%).

**Conclusions:**

The high prevalence of AMR *Salmonella* in pork and salad samples highlights the risk of transmission to humans in Kenya. These findings underscore the urgent need for strengthened food safety practices, antimicrobial stewardship and integrated AMR surveillance across livestock value chains.

## Introduction

Globally, non-typhoidal *Salmonella* (NTS) is a leading food-borne pathogen, responsible for an estimated 93.8 million cases of gastroenteritis and 155 000 deaths annually,^1^ primarily through consumption of contaminated animal source foods. The burden of NTS accounts for approximately 4.07 million disability-adjusted life years (DALYs).^2^ More concerning, antimicrobial-resistant NTS caused an estimated 214 000 deaths and 8.2 million DALYs in 2019 alone.^3^ A key contributor to this problem is the widespread use of antimicrobials in food animal production, with the pork industry being the greatest user of veterinary antimicrobials globally.^4^ This creates a risk of multidrug-resistant (MDR) *Salmonella* transmission from pigs to humans, increasing the risk of untreatable food-borne infections. A previous study conducted in the Busia County pork value chain in rural western Kenya isolated NTS from 28% of raw pork cuts, 1.9% of cooked pork and 8.6% of raw vegetable side salads served with pork. Building on these findings, this study phenotypically characterised the antimicrobial resistance profiles of 81 NTS isolates recovered from raw pork, cooked pork and accompanying salads.

## Methods

*Salmonella* spp. were isolated following International Organization for Standardization 6579-1:2017 protocols and presumptive colonies were confirmed using matrix-assisted laser desorption/ionization time-of-flight mass spectrometry as described by Gichuyia et al.^5^ Antimicrobial susceptibility testing was then performed on 81 isolates using the disc diffusion method in accordance with the guidelines of the Clinical and Laboratory Standards Institute.^6^ Briefly, standardized bacterial suspensions (adjusted to 0.5 McFarland turbidity) were cultured on Mueller–Hinton agar and exposed to a panel of antibiotics via impregnated discs. The antibiotics tested included ampicillin (10 µg), cefuroxime (30 µg), ceftriaxone (30 µg), amoxicillin–clavulanic acid (20/10 µg), nalidixic acid (30 µg), ciprofloxacin (5 µg), sulfamethoxazole/trimethoprim (1.25/23.75 µg), gentamicin (10 µg), chloramphenicol (30 µg) and tetracycline (30 µg). The control strain was *Escherichia coli* ATCC 25922. We measured zone diameters using Clinical and Laboratory Standards Institute 2018 standards, generated the multiple antibiotic resistance index (MARI) and analysed the data in R version 4.1.3 (R Foundation for Statistical Computing, Vienna, Austria).

## Results and discussion

The study found that 90% of bacterial isolates (73 of 81) were resistant to at least one antibiotic, with 22.2% classified as multidrug resistant (MDR) and 9.8% as extended drug resistant (XDR). The results were stratified by food type (raw pork, ready-to-eat pork and side salads) (Supplementary Table 1). MDR is defined as resistance to at least one agent in three antimicrobial classes, while XDR is resistance to all but two classes. Macrolides and carbapenems were not assessed; consequently, XDR estimates were interpreted conservatively. Resistance rates were notably high for gentamicin (70.4%), ampicillin (38.3%) and cefuroxime (37%).

The high level of gentamicin prevalence observed in this study is consistent with previous reports from Uganda, where *Salmonella* isolated from animal-source foods also showed elevated aminoglycoside resistance.^7^ The presence of resistant isolates in raw pork and salads suggests contamination after processing or in the environment, but the almost complete absence of MDR in cooked pork confirms the effectiveness of adequate thermal treatment. Inadequate hygiene and cross-handling increase contamination risks.^5^ All *Salmonella* isolates had a population-level mean MARI of 0.26±0.23, indicating exposure to several antimicrobials, particularly from raw pork. These findings emphasize the need for pork value chain antimicrobial stewardship and hygiene management within the pig value chain.

**Table 1.** Antibiotic antibiogram of probable *Salmonella* spp. isolates from retail pork and raw vegetables from Busia County, western Kenya.

	*Salmonella* isolates (N=81)		
Antibiotics	Resistant, n (%)	Susceptible, n (%)	Intermediate, n (%)
Ampicillin	31 (38.3)	32 (39.5)	18 (22.2)
Ceftriaxone	3 (3.7)	60 (74.1)	18 (22.2)
Amoxicillin–clavulanic acid	4 (4.9)	68 (84)	9 (11.1)
Cefuroxime	30 (37)	21 (25.9)	30 (37)
Ciprofloxacin	11 (13.6)	0 (0)	70 (86.4)
Sulfamethoxazole/trimethoprim	5 (6.2)	71 (87.7)	5 (6.2)
Gentamicin	57 (70.4)	8 (9.9)	16 (19.8)
Chloramphenicol	4 (4.9)	68 (84.0)	9 (11.1)
Tetracycline	7 (8.6)	33 (40.7)	41 (50.6)

## Conclusions

This study reveals antimicrobial-resistant NTS from the retail node within the pork value chain of Busia County. These findings reinforce the urgent need for imperative AMR surveillance, prudent antimicrobial use and hygiene improvements at slaughter and retail to effectively reduce food-borne AMR risks.

## Supplementary Material

trag019_Supplemental_File

## Data Availability

Data underlying this study are openly available at https://doi.org/10.17638/datacat.liverpool.ac.uk/3059.
