# Supplementary material for: Antimicrobial resistance patterns of Salmonella spp. identified from retail pork and raw side salads from Busia County, Kenya
Source: Trans R Soc Trop Med Hyg. 2026 Mar 6;120(6):711–3. doi: 10.1093/trstmh/trag019 (PMC13245389; doi:10.1093/trstmh/trag019)
Supplement: trag019_Supplemental_File [file trag019_supplemental_file.docx]

## **Supplementary Table 1. Prevalence of antimicrobial resistance profiles of *Salmonella* isolates**

| **Food sample type** | **Salmonella positive isolates (n)** | **No. resistant to ≥1 antibiotic** | **Drug-resistant prevalence (%) (95% CI)** | **XDR prevalence (%) (95% CI)** | **MDR prevalence (%) (95% CI)** |
| --- | --- | --- | --- | --- | --- |
| **Raw pork** | 72 | 65 | 90.3% (81.7-95.7) | 9.7% (4.0 -18.8) | 23.6% (14.4 -35.1) |
| **Cooked pork (ready to eat)** | 2 | 2 | 100% | 0 | 0 (n=2) |
| **Raw vegetables** | 7 | 6 | 85.7% (42.1-99.6) | 14.3% (27.2–39.5) | 14.3% (27.2–39.5) |
